# Arterial Damage Accompanying Supracondylar Fractures of the Humerus

**DOI:** 10.5812/kowsar.22517464.3273

**Published:** 2012-01-15

**Authors:** Mohammad Ali Mohammadzadeh, Maryam Mohammadzadeh, Ali Mohammadzadeh, Rasoul Herfatkar, Vahid Mohammadzadeh, Iraj Baghi, Hamid Heydari, Sona Najafi, Michael Jalili

**Affiliations:** 1 Department of Surgery, Gilan University of Medical Science, Rasht, IR Iran; 2 Imam Khomeini Hospital, Tehran University of Medical Science, Tehran, IR Iran; 3 Department of Radiology, Rejaee Hospital, Tehran University of Medical Science, Tehran, IR Iran; 4 Tehran University of Medical Science, Tehran, IR Iran; 5 Research Road Trauma Center4, Gilan University of Medical Science, Rasht, IR Iran; 6 Gilan University of Medical Science, Rasht, IR Iran

**Keywords:** Humerus, Fractures, Bone

## Abstract

**Background:**

Arterial damage is sometimes associated with supracondylar fractures of the humerus. Diagnosis and careful management of the fracture and arterial repair is crucial.

**Objectives:**

The aim of this study was to determine the prevalence and outcome of supracondylar fractures of the humerus with signs and symptoms of limb ischemia, before and after arterial decompression or arterial reconstruction.

**Materials and Methods:**

From September 2004 to July 2010, 225 consecutive patients with supracondylar fracture of the humerus were prospectively recruited.

**Results:**

From among 75 cases with Gartland type III fractures, 22 were found to have vascular injury.. Of the 22 cases with vascular injury, 7 patients underwent arterial reconstruction. The other 15 received arterial decompression. All patients had a satisfactory outcome.

**Conclusions:**

A high level of suspicion and careful clinical evaluation leading to an early diagnosis and management of vascular injury accompanying supracondylar fracture is very important to prevent unnecessary sequelae ranging from limb claudication, and compartment syndrome to more severe complications like Volkmann’s contracture and even limb loss.

## 1. Background

Supracondylar fracture of the humerus is the most common fracture of the elbow in the pediatric population (60%). It mostly occurs by falling on an out-stretched hand (FOOSH) (1). Due to the frequency of falls, 65-75% of all fractures in this age group affect the upper extremities (2). Not only is this type of fracture relevant because of the frequency in which it is encountered, but more importantly, because of its high complication rate seen secondary to vascular injury. The incidence of vascular injury in children after a completely displaced supracondylar fracture has been reported to be around 12%, with the brachial artery being most commonly involved (38% of all cases in Campbell et al. series) (3, 4). Arterial injury ranges anywhere from vascular contusion and intimal damage, to complete arterial transection (5, 6). The dreaded vascular complications include brachial artery occlusion and possible limb loss, compartment syndrome or, Volkmann’s contracture (1). Another possible long-term vascular complication is limb claudication (due to inadequate repair of the brachial artery or distal thrombus migration, with impeded blood flow) (2). Non-vascular complications include deformity and neurologic injury (median, ulnar, and radial nerves). The anterior interosseous nerve, a branch of the median nerve being most commonly involved (20-45% of all nerve injuries) (1). Prevention of these complications depends largely on a timely diagnosis, aggressive treatment, and a high index of suspicion in a child with a suggestive history and physical exam. In evaluation of supracondylar fractures of the humerus, the physician must pay close attention to the neurovascular exam (1). Failure to assess a distal pulse in a patient may result in the loss of thechild’s limb. It is important to realize that by the time a patient develops pain, paresthesia, pallor, poikilotherma, or paralysis of an affected limb, muscle necrosis has already begun, and may be too late for limb salvage. Early diagnosis and prompt management of the fracture and arterial injuries is mandatory to prevent these and disabling complications (4).

## 2. Objectives

The aim of this study was to determine the prevalence and outcome of supracondylar fractures of the humerus with signs and symptoms of limb ischemia, before and after arterial decompression or arterial reconstruction.

## 3. Materials and Methods

We assessed 225 consecutive patients with a diagnosis of humeral supracondylar fracture from September 2004 to July 2010. Age, sex, cause, time of injury, type of fracture, and vascular status (signs and symptoms of arterial insufficiency) were recorded. Signs of ischemia were assessed by palpation of radial, ulnar, and brachial pulses, finger mobility and muscle tonicity, capillary refill of nail beds, puller pulse oximetery and hand temperature, were carefully recorded.

Emergent open reduction and internal fixation of the fractured humerus was performed along with a thorough exploration of neurovascular structures by aid of an anteromedial incision in 22/225 cases. Fifteen of the 22 cases presenting with ischemia underwent arterial decompression along with fracture site open reduction and internal fixation. The remaining 7 cases were found to have intimal damage and thrombosis of the brachial artery necessitating arterial reconstruction or interposition graft placement in addition to open reduction/ fixation of humeral fracture. A successful outcome was considered as a palpable distal pulse and viable, warm extremity 24 hours post-op.

## 4. Results

In the patient population of 225 patients with supracondylar fracture there were 95 males and 130 females (42% and 58%, respectively). The mean age was (8±3) years, with the range between 2-15 years. The type of supracondylar fracture of the humerus was simple (Gartland type I) in 32 cases (14.22 %); 118 cases (52.4%) were minimally to moderately displaced (Gartland type II) ; and 75 cases (33.2%) were severely displaced (Gartland type III) fractures (Table 1).

**Table 1.** Relationship of Gartland Fracture Type with Vascular Injury, Need for Surgical Exploration, and Long Term Sequelae

Gartland Fracture Type	Presenting Fracture	Associated Vascular Injury	Need For Surgical Exploration	Arterial Exploration/ Decompression	Arterial reconstruction/ Interposit-ion graft	Post-Fracture and/or Surgical Complications
I ^a^	32 (14.2%)	0	0	0	0	0
II ^b^	118 (52.4%)	0	0	0	0	0
III ^c^	75 (32.5%)	22 (24%)	22 (24%)	15/22 (68.1%)	5/7 (71.4%)1 2/7 (28.6%)2	0
TOTAL	225	22/225 (9.8%)	22/225 (9.8%)	15/225 (6.7%)	7/225 (3.1%)	0

^a^ Non-displaced

^b^ Minimal to moderately displaced

^c^ Severely displaced

Of the 225 patients with supracondylar fracture, 22 cases presented with signs and symptoms of acute ischemia in the hand. In 75 of the 225 total cases, severe dislocation of the fractured site (Gartland type III) was noted. Of the 75 Gartland type III fractures, 22 cases resulted in vascular compromise of the distal extremity (24%). This translates to a 9.8% risk for vascular compromise secondary to supracondylar fracture of the humerus in our study (22/225). In 15 cases with vascular compromise, exploration of the brachial artery followed by arterial decompression resulted in positive distal pulses in all 15 cases. Achievement of arterial patency in 7 patients found to have arterial thrombosis and intimal damage of the brachial artery was accomplished by arterial reconstruction in 5 cases and interposition grafts in the remaining 2. Interposition saphenous vein graft with primary resection and reanastamosis was performed in the 2 latter cases. All 7 cases were successfully treated in our study with a favorable outcome confirmed by palpable distal radial artery pulses, accompanied by warm, functional extremities.

## 5. Discussion

Vascular injuries secondary to supracondylar fractures of the humerus need emergency orthopedic and vascular management. Acute ischemic findings associated with elbow fractures include severe extremity edema and pain which make diagnosing the vascular injury that much more difficult (7). As a result these patients are usually referred with a delay in diagnosis and signs of contracture and frank ischemia. Initially after trauma of the elbow, surrounding ecchymosis associated with joint deformity should be carefully and promptly evaluated (Gartland type I). Ischemic Volkmann’s contracture and hand necrosis with severe dislocation of the elbow are late signs often revealing mismanagement or misdiagnosis of the patient (5). Pain, paresthesia, paralysis, pulslessness, pallor or poikilothermia when apparent are associated with muscular necrosis (8). However, they may be noted early and can be helpful in guiding the management of vascular injury (9).

Color Doppler ultrasonography for evaluation of arterial patency and circulation at the fracture site is useful. Arteriography in those patients with debatable vascular injury is indicated in order to localize the vascular injury, (10). Some authors like Shaw et al emphasized that exploration without angiography in suspected cases is mandatory (8) and that arteriography does not provide further information regarding the site of vascular damage (4). In our study, while simultaneously reducing the fracture and exploring the suspected damaged artery, we routinely successfully managed the arterial injury without wasting precious time in performing angiography. We did not have angiography readily available, and because of the length of time it usually took to coordinate with other services, it was not seen as an procedure in suspicious cases of arterial injury.

**Figure 1.**
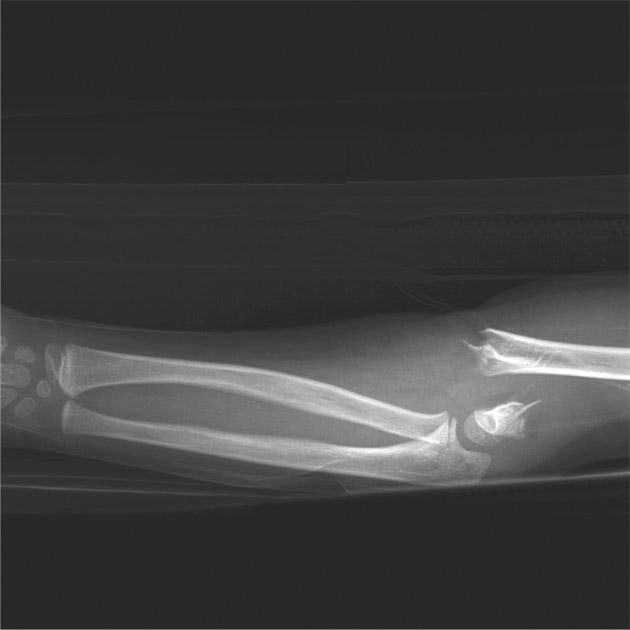
Supracondylar fracture Gratland III

Based on our criteria, among the 225 patients with supracondylar fractures, 22 out of 75 patients in addition to having combined fractures and dislocations (Gartland type III), were also complicated by vascular injury; 7 cases with arterial injury were treated with vascular reconstruction while the remaining 15 underwent arterial decompression. It is important to point out that the underlying cause of arterial insufficiency in a small number of cases presenting with supracondylar fracture/ dislocation may simply be arterial spasm (1). Although less invasive treatment by closed reduction and release of the entrapped artery, in retrospect, may be the better choice; immediate surgery does not seem to cause any additional morbidity (7). Rabee et al. in their study showed that in most cases studied in which the limb was not threatened, they found that early exploration allowed assessment of neurovascular structures and type of injury with the additional advantage of decompression of both the antecubital fossa and flexor compartment syndrome. The fracture could also be reduced directly under vision, avoiding further damage to the neurovascular bundle. Follow up of cases revealed good palpable radial pulse in all cases (2). Also worth mentioning is the special circumstance when a child presents with a pulseless, but otherwise well-perfused hand. Debate exists that if the pulse remains absent after closed reduction but clinically the hand remains warm, (4) arterial exploration may not be necessary as elbow edema/ arterial spasm are again theorized to be responsible for this phenomenon (1). Following all procedures in our study, all patients developed good perfusion and amputation of the affected limb was never found to be necessary. In Kumar et al. study frank gangrene and amputation in one out of four patients with vascular compromise occurred; we did not have any limb gangrene (Figure 1). It was reported that the case requiring amputation in the previously mentioned study was referred to their institution after 7 days had elapsed since the injury (7). The interval between injury and treatment for our study was 4-20 hours. We noticed that in children with arterial damage accompanying supracondylar fracture, a longer time is required to manage acute ischemia.

At 30 day follow-up all patients showed good results with positive palpable distal pulses, and warm, mobile extremities. In a similar study, Ottolenghi revealed that after a longer time delay of more than 24 hours, arterial reconstruction also showed encouraging results (5). Shaw and his group presented their cases of arterial damage with supracondylar fractures without using Doppler sonography or angiography with the same favorable results as in our study (8). Arterial damage accompanying a supracondylar fracture requires immediate revascularization (arterial release or reconstruction). Clinical examination and, particular attention to signs of vascular compromise in the affected extremity is crucial. Time is invaluable, and in areas where angiography is time consuming or technically not feasible, we have found it to be better to proceed straight to revascularization while performing an open reduction, as the unnecessary test provides no significantly useful information to the surgeon.
